# Efficacy and Safety of Abiraterone Acetate and Enzalutamide for the Treatment of Metastatic Castration-Resistant Prostate Cancer: A Systematic Review and Meta-Analysis

**DOI:** 10.3389/fonc.2021.732599

**Published:** 2021-08-27

**Authors:** ZhenHeng Wei, ChuXin Chen, BoWen Li, YongYue Li, Hong Gu

**Affiliations:** ^1^Inner Mongolia Medical University, Hohhot, China; ^2^Peking Union Medical College Hospital, Beijing, China; ^3^Inner Mongolia Medical University, Rehabilitation Department of Baotou Steel Hospital, Baotou, China; ^4^Inner Mongolia Baotou Steel Hospital, The Third Clinical Medical College of Inner Mongolia Medical University, Baotou, China

**Keywords:** abiraterone acetate, enzalutamide, mCRPC, meta-analysis, systematic review

## Abstract

**Objective:**

The androgen receptor-targeting drugs abiraterone acetate and enzalutamide have shown positive results as treatments for metastatic castration-resistant prostate cancer (mCRPC). Therefore, a meta-analysis was conducted to compare the efficacy and safety of abiraterone acetate and enzalutamide in patients with mCRPC.

**Methods:**

We retrieved relevant articles from PubMed, Cochrane, and EMBASE published before December 31, 2020. Eleven articles were initially selected, and four phase III, double-blind, randomized controlled trials of abiraterone acetate and enzalutamide that involved 5199 patients with mCRPC were included. The end points were time to prostate-specific antigen progression (TTPP), according to the prostate-specific antigen working group criteria; overall survival (OS); and radiographic progression-free survival (rPFS).

**Results:**

Four randomized, controlled clinical trials involving 5199 patients were included in this study. The results of the meta-analysis showed that compared with placebo alone, abiraterone significantly improved OS (HR=0.69, 95% CI: 0.60-0.8, P<0.00001), rPFS (HR=0.64, 95% CI: 0.57-0.71, P < 0.00001), and TTPP (HR=0.52, 95% CI: 0.45-0.59, P < 0.00001) in patients with mCRPC. Compared with placebo, enzalutamide significantly improved OS (HR=0.67, 95% CI: 0.59-0.75, P<0.00001), rPFS (HR=0.33, 95% CI: 0.29-0.37, P< 0.00001), and TTPP (HR=0.19, 95% CI: 0.17-0.22, P < 0.00001). An indirect comparison was performed to compare the efficacy of abiraterone and enzalutamide. The results showed that there was no significant difference between abiraterone and enzalutamide with regard to improving the OS of patients with mCRPC (HR=1.03, 95% CI: 0.854-1.242). Enzalutamide was superior to abiraterone with regard to improving rPFS in patients with mCRPC (HR=0.516, 95% CI: 0.438-0.608). With regard to improving TTPP, the efficacy of enzalutamide was better than that of abiraterone (HR=0.365, 95% CI: 0.303-0.441). In sAE, there was no difference between abiraterone and enzalutamide (P=0.21, I^2 =^ 38%).

**Conclusions:**

Compared with placebo, both abiraterone and enzalutamide significantly prolonged OS, rPFS, and TTPP in patients with mCRPC. There was no difference in safety between abiraterone and enzalutamide. In addition, enzalutamide had better efficacy than abiraterone with regard to improving rPFS and TTPP but not OS, but the level of evidence was low. Therefore, a large direct comparison trial is needed to compare the efficacy of the two drugs.

**Systematic Review Registration:**

PROSPERO, identifier (CRD42021226808)

## Introduction

Prostate cancer became the leading cause of new cancer cases in the United States and the second leading cause of all cancer-related deaths in 2020, according to Cancer Statistics (1). Advanced prostate cancer has a poor prognosis and is difficult to treat. It can develop into castration-resistant prostate cancer (CRPC) within (1 to 2) years and readily progresses to metastatic CRPC (mCRPC) (2). In recent years, advances in therapeutic drugs have begun to change the treatment philosophy and strategies for this stage of the disease, although high-level evidence is still lacking. In addition, there are still controversies about how to rationally select and use different drugs to improve the overall treatment efficacy (3–7).

Abiraterone acetate is an enzyme inhibitor of CPY17 that inhibits the residual synthesis of androgen after androgen deprivation therapy and can be used for the treatment of mCRPC in patients who have previously received chemotherapy (8, 9). Enzalutamide (MDV3100), an oral drug targeting the androgen receptor signalling pathway, can competitively inhibit androgen receptor binding. Compared with antiandrogen drugs, such as bicalutamide, previously used in clinical therapy, enzalutamide has a 5- to 8-fold greater affinity for the androgen receptor (10, 11). When used for the treatment of advanced or metastatic hormone-sensitive prostate cancer, both abiraterone acetate and enzalutamide have been shown to reduce mortality and improve overall survival (OS) (12–14). It is necessary to maximize the efficacy of drug therapy for mCRPC and improve the OS of patients (6, 15, 16). Therefore, we conducted a meta-analysis to evaluate the efficacy and safety of these two drugs in patients with mCRPC.

## Materials and Methods

We registered the protocol for this systematic review with PROSPERO (CRD42021226808). This report complies with the PRISMA meta-analysis extension statement. The trials covered in this article were registered on internationally recognized clinical trial registries, such as the North American Clinical Trial Registry (www.clinicaltrials.gov). In addition, only clinical data of patients are collected in this paper, without intervention in the treatment plan of patients, which will not bring physiological risks to patients, so there is no need for ethical review.

### Search Strategy

We retrieved relevant studies from PubMed, Cochrane Library, EMBASE, and ClinicalTrials.gov from the date of database inception to December 31, 2020. MeSH terms and keywords such as “prostate cancer,” “abiraterone,” “enzalutamide,” “clinical trials as topic” and relevant variants were used. For example, the following search terms were used in PubMed: ((((((((“Abiraterone Acetate”[Mesh]) OR (17-(3-pyridyl)-5,16-androstadien-3beta-acetate)) OR (Zytiga)) OR (CB 7630)) OR (CB-7630)) OR (CB7630)) OR ((((Enzalutamide) OR (MDV3100)) OR (MDV-3100)) OR (enzalutamide))) AND ((clinical[tiab] AND trial[tiab]) OR “clinical trials as topic”[mesh] OR “clinical trial”[pt] OR random*[tiab] OR “random allocation”[mesh] OR “therapeutic use”[sh])) AND ((((((((((((((((((“Prostatic Neoplasms”[Mesh]) OR (Prostate Neoplasms)) OR (Neoplasms, Prostate)) OR (Neoplasm, Prostate)) OR (Prostate Neoplasm)) OR (Neoplasms, Prostatic)) OR (Neoplasm, Prostatic)) OR (Prostatic Neoplasm)) OR (Prostate Cancer)) OR (Cancer, Prostate)) OR (Cancers, Prostate)) OR (Prostate Cancers)) OR (Cancer of the Prostate)) OR (Prostatic Cancer)) OR (Cancer, Prostatic)) OR (Cancers, Prostatic)) OR (Prostatic Cancers)) OR (Cancer of Prostate)).

### Study Selection

We included phase III, randomized, double-blind, placebo-controlled clinical trials of the use of abiraterone or enzalutamide for the treatment of mCRPC. Nonrandomized controlled studies, studies with poor experimental designs, studies with inconsistent outcome indicators, reviews, systematic reviews, case reports, studies with median estimates reported without confidence intervals or boundary values, and studies with no usable clinical results were excluded.

### Data Extraction

The literature search and screening process was conducted by two investigators independently in accordance with the established inclusion and exclusion criteria. Any differences were resolved by the other investigator. The following data were extracted: year of publication, number of subjects, duration of the intervention, and main outcome indicators. If necessary, the authors were emailed to obtain data on indicators not reported in the study.

### Outcomes

The primary outcome was OS, and the secondary outcomes were radiographic progression-free survival (rPFS), time to prostate-specific antigen development (TTPP) and serious adverse events (sAEs). OS was defined as the time from the date of randomization to the date of death from any cause. rPFS was defined as the time from randomization to the earliest objective evidence of radiographic progression or death due to any cause. TTPP was defined as the time from randomization to the occurrence of the first bone-related event. An adverse event that results in death is life-threatening, requires inpatient hospitalization or extends a current hospital stay, the results in an ongoing or significant incapacity, interferes substantially with normal life functions, or causes a congenital anomaly or birth defect. Medical events that do not result in death, are not life-threatening, or do not require hospitalization may be considered serious adverse events if they put the participant in danger or require medical or surgical intervention to prevent one of the results listed above.

### Quality Assessment

The Cochrane risk of bias assessment tool was used, which addresses random sequence generation, allocation concealment, blinding methods (double blind, triple blind), the integrity of outcome data, the selective reporting of study results, and other sources of bias. Quality assessments were performed, and each indicator was assessed as a “low risk of bias”, “high risk of bias” or “unclear”. The following types of bias were considered: selection bias, performance bias, detection bias, attrition bias, reporting bias, and other bias.

### Statistical Analysis

RevMan 5.3 and Indirect Treatment Comparison (ITC) software were used in this meta-analysis to perform indirect comparisons of the use of abiraterone and enzalutamide for the treatment of mCRPC. The hazard ratio (HR) was used as the efficacy effect estimate, and the 95% confidence interval (CI) was calculated. The studies that reported the HR specified whether it was obtained through the Parmar and Tierney method, p-value estimation or survival curve analysis.

The Cochrane Q test was used to assess the heterogeneity of the included studies (the significance level was α=0.1). The magnitude of the heterogeneity was determined quantitatively. If the P value of the Q test was > 0.1 and I² was < 50%, it suggested that there was limited heterogeneity, and a fixed-effect model was used for the meta-analysis. If the P value of the Q test was ≤0.1 or I² was > 50%, sensitivity analyses or subgroup analyses were needed to explore the source of heterogeneity. After removing the influence of heterogeneity, a fixed-effect model was used for the meta-analysis. The significance level for the meta-analysis was set at α=0.05.

## Results

### Literature Search

**Figure 1:**
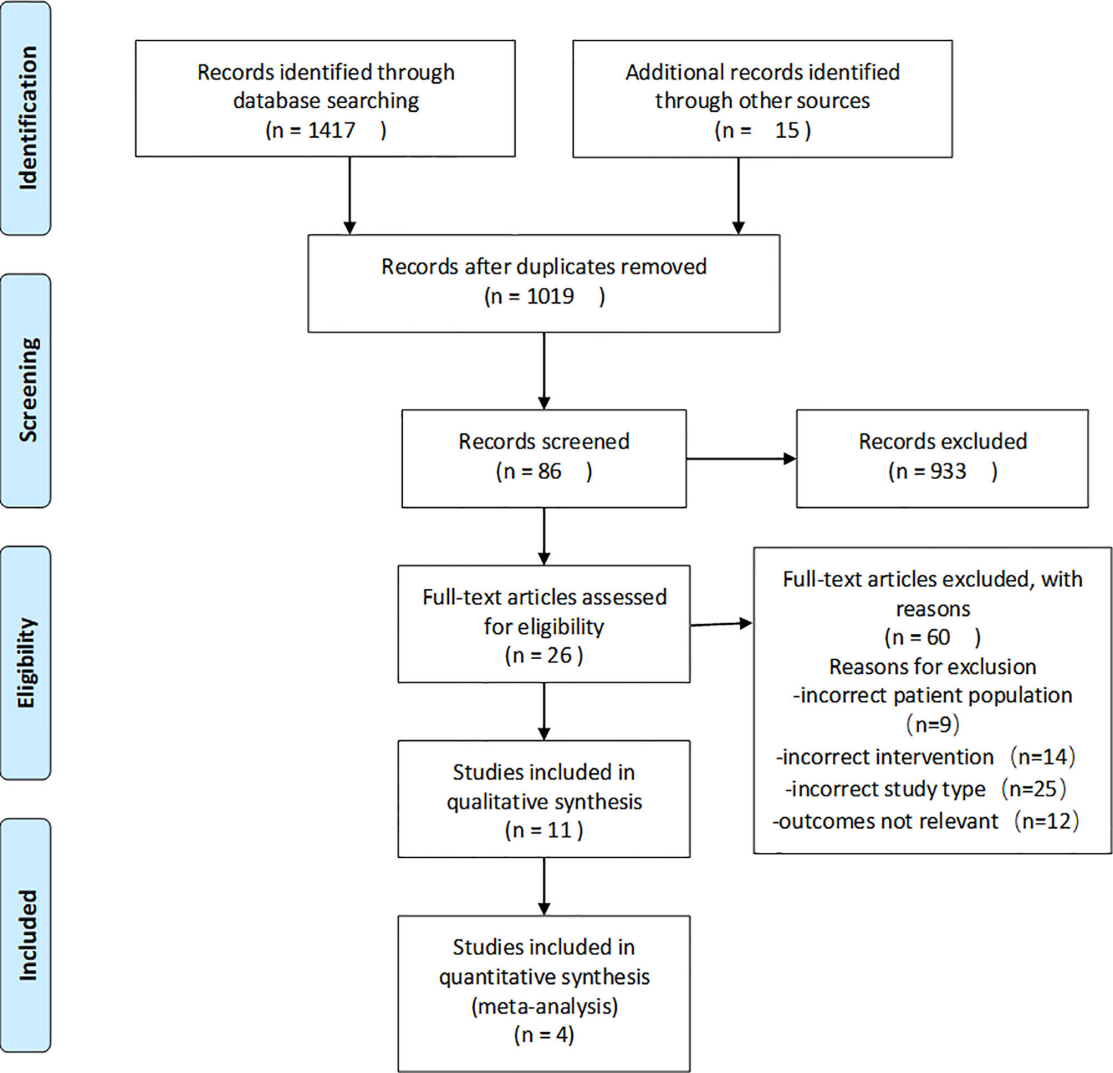
Flowchart for the systematic review process and data acquisition.

The results of the search and screening process are shown in **Figure 1** in the form of a flow diagram. A total of 1,417 papers were initially retrieved from the electronic databases, and 15 papers were obtained by other methods. A total of 413 duplicate articles were excluded, and 933 were excluded based on their titles and abstracts. Twenty-six articles were read in full, and 4 clinical trials were selected from the remaining 86 articles.

The study included 4 clinical trials (7, 17–19), all of which were published in English and administered a placebo to the control group. All were phase III, double-blind, randomized controlled trials. Each trial included participants from multiple countries and regions. The racial and regional bias in each experiment was smaller, which will not affect the results and conclusions. Abiraterone was used for the treatment of mCRPC in 2 clinical trials (2283 patients), and enzalutamide was used in 2 clinical trials (2916 patients). **Table 1** summarizes the characteristics of the included studies and the key baseline patient characteristics. The results from the 4 clinical trials were reported as OS, rPFS, TTPP and sAE. Refer to **Table 2** for details.

**Table 1:** Characteristics of the eligible studies.

Study	Years	NCT Number	Pahse	Line	Masking	OS follow-up	Patients	Treatment (N)	Control (N)	Median Age (SD)	region
Karim Fazzi 2012	2008-2014	00638690	3	2	Quadruple	Up to 60 months	1187	abiraterone + prednisone (797)	prednisone + placebo (390)	69 (8.46)	multicenter
Kurtr Miller 2017	2009-2018	00887198	3	1	Quadruple	Up to 61 months	1088	abiraterone + prednisone (546)	prednisone + placebo (542)	70.3 (8.76)	multicenter
Andrew J 2020	2009-2018	00974311	3	2	Triple	up to 101 months	1199	Enzalutamide (800)	placebo (399)	68.7 (8.11)	multicenter
Nancy Devlin 2017	2010-2020	01212991	3	1	Triple	up to 3 years	1717	Enzalutamide (872)	placebo (845	71.3 (8.47)	multicenter

**Table 2:** Indirect comparative results of abiraterone acetate and enzalutamide.

Study ID	OS Median (95% CI)		TTPP Median (95% CI)		rPFS Median (95% CI)		sAE RR (95% CI)
	Experimental	Placebo Comparator	Experimental	Placebo Comparator	Experimental	Comparator	
Fizaziki K. *(17)	450.0	332.0	309.0	200.0	171.0	110.0	1.04 (0.91, 1.19)
	(430.0 to 470.0)	(310.0 to 366.0)	(255.0 to 421.0)	(170.0 to 254.0)	(169.0 to 192.0)	(88.0 to 168.0)	
Miller K. 2017	34.66	30.29	11.07	5.55	NA	8.28	1.4 (1.18, 1.66)
	(37.72 to 36.80)	(28.65 to 33.28)	(8.51 to 11.24)	(5.39 to 5.59)	(11.66 to NA)	(8.12 to 8.54)	
Devlin N. (7)	32.4	30.2	11.2	2.8	NA	3.9	1.62 (1.41, 1.86)
	(30.1 to NA)	(28.0 to NA)	(11.1 to 13.7)	(2.8 to 2.9)	(13.8 to NA)	(3.7 to 5.4)	
Armstrong A. J. (19)	18.4	13.6	8.3	3.0	8.3	2.9	1.03 (0.88. 1.19)
	(17.3 to NA)	(11.3 to 15.6)	(5.8 to 8.3)	(2.9 to 3.7)	(8.2 to 9.4)	(2.8 to 3.4)	

Not Assement (NA).

*The statistical unit of the study is day.

### Quality of the Included Studies

**Figure 2:**
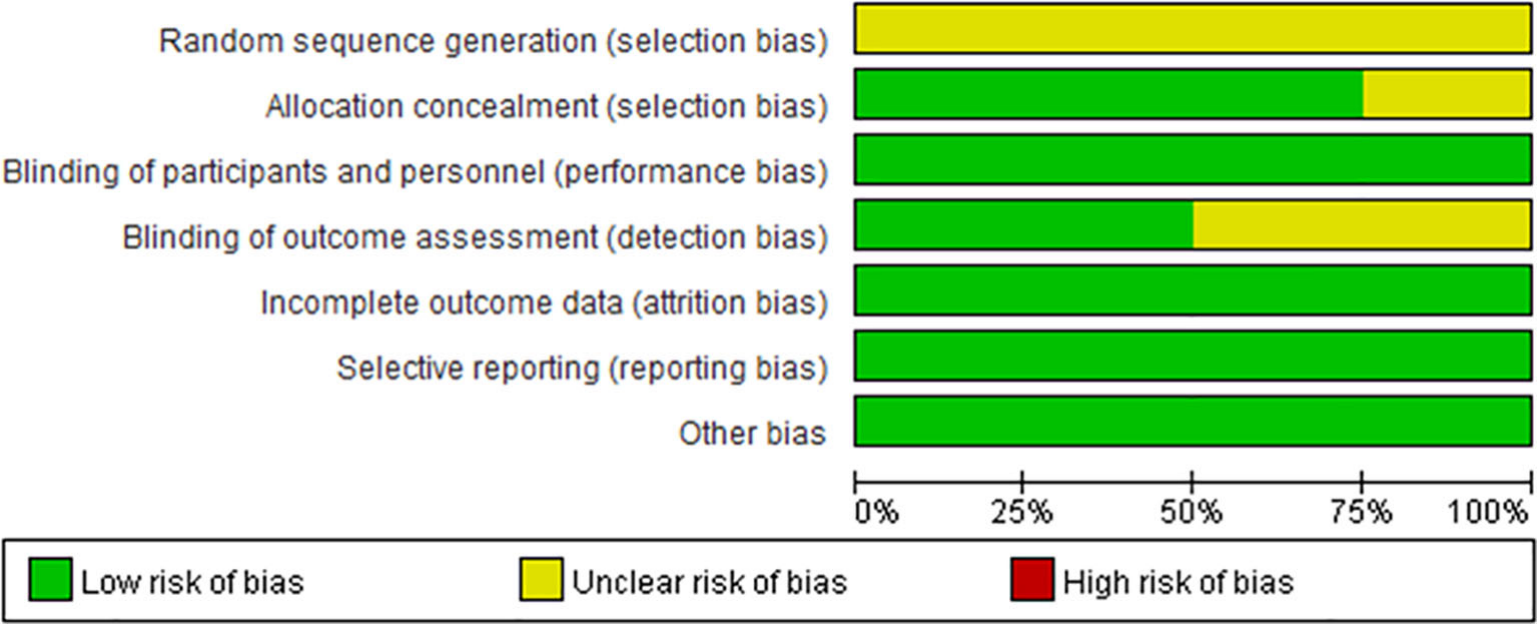
Risk of bias summary. Green circles represent a low risk of bias; red circles represent a high risk of bias; yellow circles represent an unknown risk of bias.

**Figure 3:**
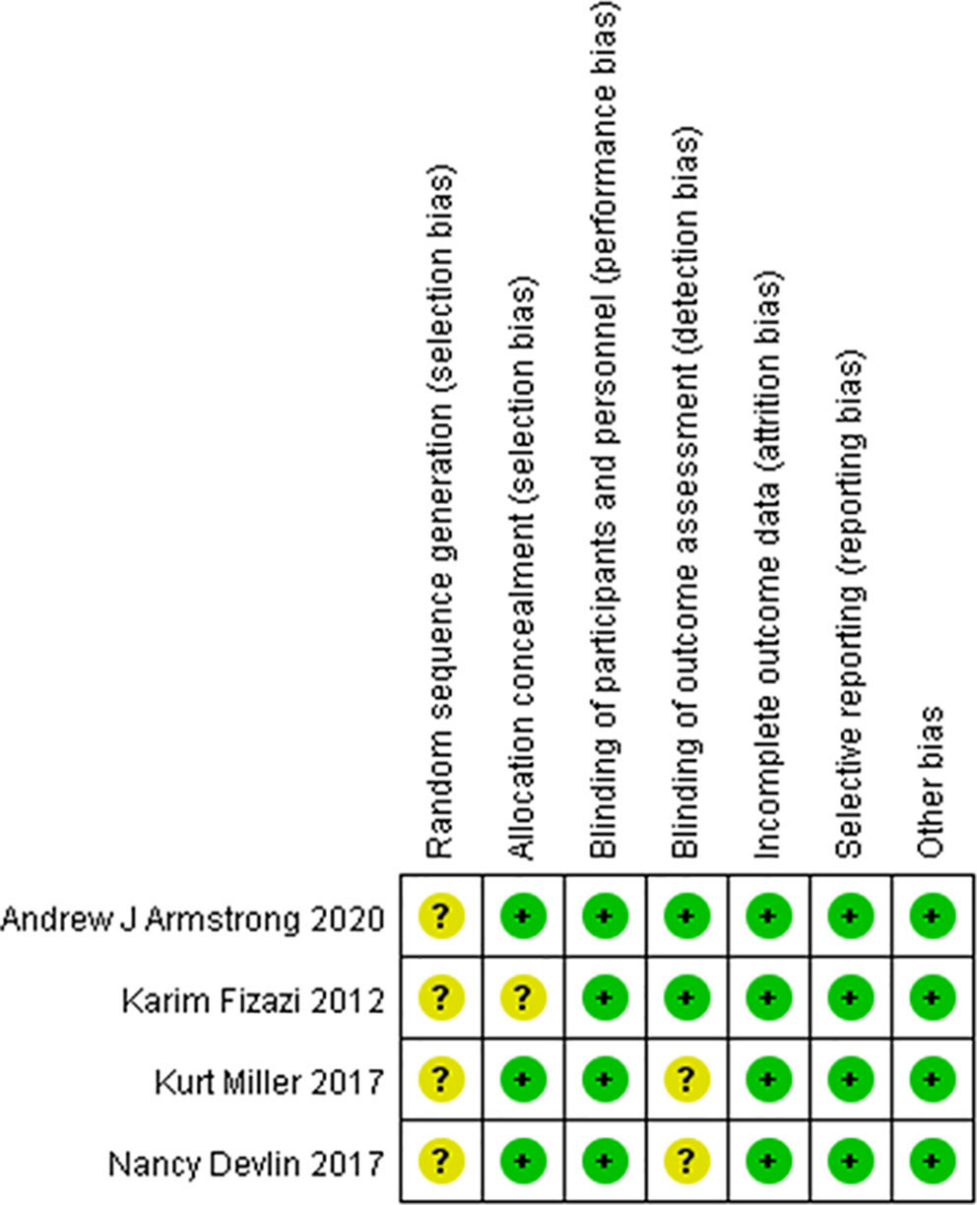
Risk of bias summary. Green circles represent a low risk of bias; red circles represent a high risk of bias; yellow circles represent an unknown risk of bias.

The risk of bias was evaluated in the four included clinical trials. All four trials were conducted with blinding of the participants, investigators, and outcome assessors. Data for each of the major outcome indicators were reported. Although random assignment was performed, the methods were not described in detail. The results were visualized with RevMan 5.3, as shown in **Figures 2**, **3**.

### Overall Survival

**Figure 4:**
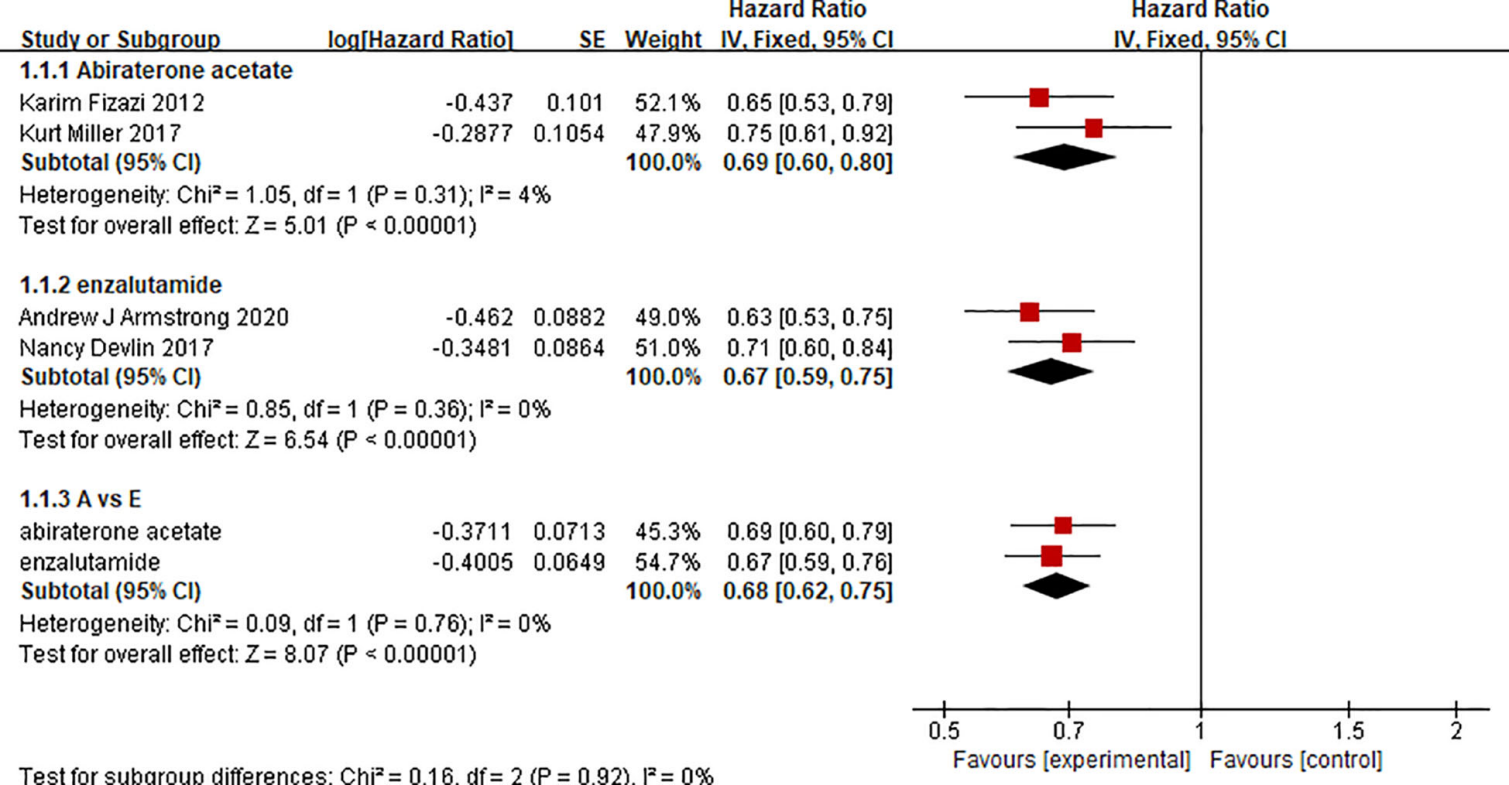
Forest plots for Overall survival in studies.

There were 2 clinical trials comparing the effect of abiraterone with that of a placebo on the OS of 2283 mCRPC patients. Two clinical trials compared the effect of enzalutamide with that of a placebo on the OS of 2283 mCRPC patients. The test of heterogeneity indicated that there was limited heterogeneity between the studies (I^2 =^ 4%, P=0.31; I^2 =^ 0%, P=0.36), and the fixed-effect model was used for the meta-analysis. The results showed that abiraterone and enzalutamide had significant advantages over placebo with regard to the OS of mCRPC patients (HR=0.69, 95% CI: 0.60-0.80; HR=0.67, 95% CI: 0.59-0.75; Z=5.01, P < 0.00001; Z=6.54, P < 0.00001) (**Figure 4**). Further indirect comparisons based on different treatment regimens showed no difference between abiraterone and enzalutamide (HR=1.03, 95% CI: 0.854 – 1.242) with regard to OS in mCRPC patients.

### Time to Prostate-Specific Antigen Progression

**Figure 5:**
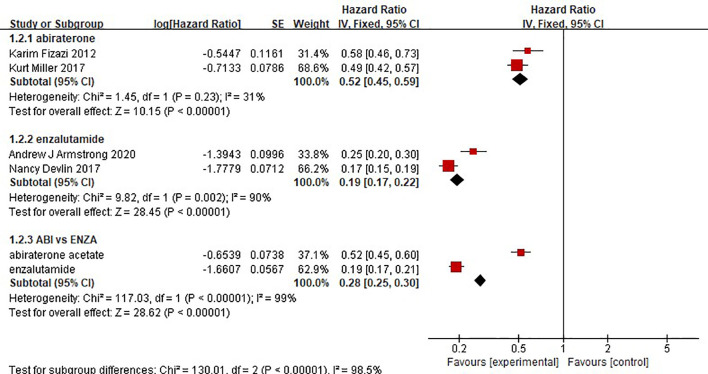
Forest plots for time to prostate-specific antigen progression in studies.

The results showed that abiraterone had a significant advantage over the placebo regarding TTPP in mCRPC patients (HR=0.52 95%CI:0.45 to 0.59). Enzalutamide also resulted in a significantly longer TTPP in mCRPC patients than placebo (HR=0.19, 95% CI: 0.17-0.22) (**Figure 5**). The test for heterogeneity suggested that the abiraterone subgroup had moderate heterogeneity (I^2 =^ 31%, P < 0.00001). However, the test for heterogeneity indicated that there was considerable heterogeneity in the enzalutamide subgroup (I^2 =^ 90%, P < 0.00001). The heterogeneity stemmed from differences in observation time and the number of placebo groups between the two clinical trials. The indirect comparison showed that enzalutamide had a greater effect on TTPP in mCRPC patients than abiraterone (HR=0.365, 95% CI: 0.303-0.441).

### Radiographic Progression-Free Survival

**Figure 6:**
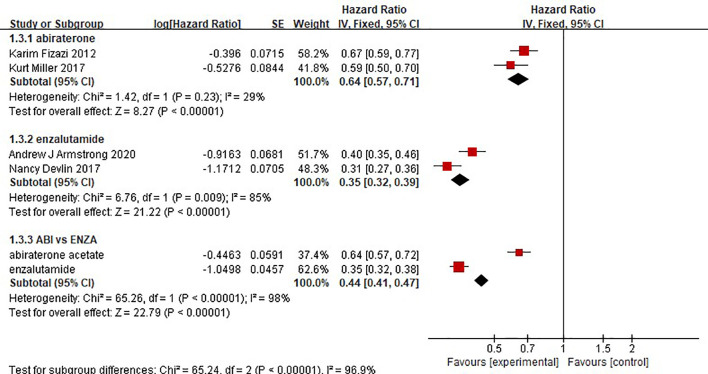
Forest plots for radiographic progression-free survival in studies.

Two clinical trials each compared the effect of abiraterone or enzalutamide with that of a placebo on the rPFS of 5199 mCRPC patients. The results showed that abiraterone had a significant advantage over the placebo regarding rPFS in mCRPC patients (HR=0.64, 95% CI: 0.57-0.71; Z=8.27, P < 0.0001) (**Figure 6**). The test for heterogeneity suggested that the abiraterone subgroup had moderate heterogeneity (I^2 =^ 29%, P < 0.00001). However, there was considerable heterogeneity in the enzalutamide subgroup (I^2 =^ 85%, P < 0.00001). The heterogeneity stemmed from differences in observation time and the number of placebo groups between the two clinical trials. Compared with placebo, enzalutamide significantly improved the rPFS of mCRPC patients (HR=0.35, 95% CI: 0.32 - 0.39). The indirect comparison showed that enzalutamide had a greater effect than abiraterone on the rPFS of mCRPC patients (HR=0.547, 95% CI: 0.472-0.634).

### Serious Adverse Event

**Figure 7:**
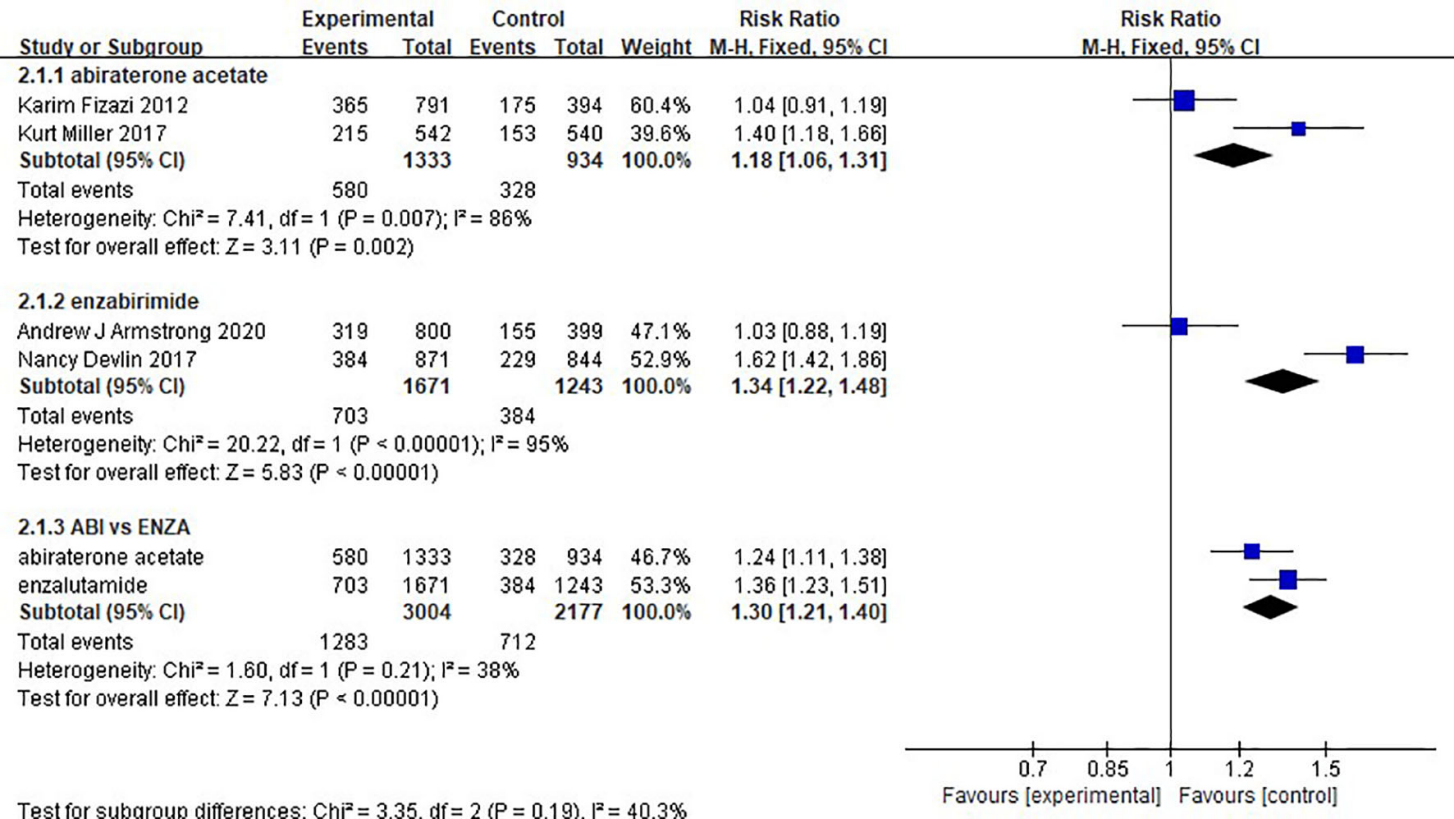
Forest plots for serious adverse events in studies.

There was no difference in safety between abiraterone and enzalutamide. (1.18, 95% CI: 1.06-1.31; 1.34, 95% CI: 1.22-1.48). The test of heterogeneity indicated that there was limited heterogeneity between the studies (I^2 =^ 38%, P=0.21) (**Figure 7**).

## Discussion

By calculating and combining HR, our results show that both enzalutamide and abiraterone improve patients’ OS compared with placebo. In addition, enzalutamide was more effective in improving TTPP and rPFS than abiraterone acetate and prednisone/prednisolone combination therapy. There was no significant difference in safety between the two drugs. This finding is significant because it provides substantial evidence for clinical studies in patients with metastatic prostate cancer.

Unlike the study by Wang et al (20) using OR to evaluate the effectiveness of two things, an advantage of this study is the use of combined HR to evaluate the efficacy of AR inhibitors. Compared to the median values for OS, rPFS, and TTPP, HR takes into account both time and queue size (21–23). However, when comparing security, we used RR to compare sAE because we could not obtain more details of the data. In addition, since there have been a large number of meta-analyses comparing abiraterone and enzalutamide, few meta-analyses have included all relevant randomized clinical trials (RCTSs) and directly combined the hazard ratio (HR) of overall survival (OS) and progression-free survival (PFS) for the two drugs. Additionally, in the absence of large RCTs for direct comparison, we used both subgroup analyses and indirect comparisons to evaluate the efficacy and safety of the drugs. Moreover, the study by Wang et al (20) contains multiple studies, but not every study has a sufficient sample size, and there may be regional bias. The clinical trials in this study have a large sample size and come from multiple regions, and the regional and ethnic bias may be smaller. Therefore, the conclusion of this study may be more convincing. First, in the subgroup analysis, there was some heterogeneity in the abiraterone group and the enzalutamide group, which may be caused by the different inclusion criteria of the experiment. In the study by Fizazi K et al (24), patients with mCRPC confirmed histologically or cytologically were eligible if they had previously received docetaxel and had received up to two previous chemotherapy treatments; however, in the study by Miller, K., et al (18), patients must not have received chemotherapy before. Second, another source of heterogeneity may be that the severity of disease and initial PSA levels were not exactly the same in the two studies. In addition, follow-up times were different between the two studies. All of these factors would result in heterogeneity within the abiraterone group. In the study by Devlin, N et al. (7, 25), on the other hand, patients had to have never received cytotoxic chemotherapy; however, in the study by Armstrong, A.J et al. (19, 26, 27), patients needed to be treated with one or two advanced chemotherapy regimens, and at least one regimen had to contain docetaxel. The intragroup heterogeneity of enzalutamide may be derived from this. However, the OS heterogeneity of the two subgroups was very small, so the heterogeneity could also be derived from statistics. The results of the meta-analysis were convincing despite the heterogeneity of the multiple aggregate results. For rPFS, the study by McCool.R et al. (28) uses the method of network analysis to evaluate. However, many comparisons are made through only one experiment, and the sample size of some experiments is too small, and there may be large heterogeneity among the subgroups of the network analysis. As the number of hypothesis tests increases, the rate of false positive errors will increase substantially, even if there is no difference in effect. For the comparison of two drugs, indirect comparison is a better choice. But the conclusion is similar. Enzalutamide is better than abiraterone in improving rPFS.

In addition, we comprehensively explored the safety of abiraterone and enzalutamide and found that AR inhibitors resulted in a higher overall incidence of AE, actually significantly reduced the incidence of high-grade AE, and similar rates of AE leading to death or withdrawal. All patients in the Karin and Kurt trials were assigned to the mandatory use of prednisone to avoid or mitigate adverse events associated with related mineral corticosteroids (18, 24, 29). In contrast, in the Andrew J and Nancy trials (19, 26, 27), enzalutamide did not require the use of prednisone. The safety of abiraterone and enzalutamide appears to be acceptable and manageable, as these AEs can be managed through appropriate medical monitoring. It is important to note that heterogeneity exists between studies, which may be due to differences and heterogeneity in the treatment of abiraterone and enzalutamide. Given the limitations of the study’s reliance on published study results rather than original individual patient data, some important baseline characteristics of patients, namely age, bone injury, visceral disease, ECOG performance status score, and GS, may also play a key role in this large heterogeneity. In addition, the incidence of high-level adverse events (sAEs) in the AR inhibitor group *versus* the control group was not adequately compared. To reduce potential bias, we only extracted data that strictly met our inclusion criteria, resulting in many AEs being excluded from the analysis. Therefore, in future clinical practice, AR inhibitors should be considered an effective and safe treatment option for patients with CRPC, although practitioners should pay particular attention to the AEs mentioned in our study, especially the high-level AEs. In addition, the use of uniform AE reporting standards for further in-depth data analysis is of great significance to the researchers who carried out the original study.

In the current study, it was significant and interesting for us to determine whether abiraterone acetate and enzalutamide obtained any different benefits in mCRPC through subgroup analysis. This will provide evidence for drug selection in clinical treatment. However, some questions remain unanswered: the most appropriate patient population, potential cross-resistance mechanisms, optimal sequential administration, and possible combination strategies.

There were limitations of this study, such as the limitation of the included studies to those published in English. The references of the included studies were not evaluated, which may have led to the omission of relevant studies. The use of random allocation was described, but the details of the random allocation and the allocation concealment were not included. When extracting the data, some studies did not directly report the effect size and the corresponding confidence interval; therefore, statistical methods were used to calculate the effect size based on the available information, which may have resulted in slightly different results. There are some differences between the results of direct and indirect comparisons; therefore, more prospective studies are needed to verify the findings.

## Conclusions

In summary, the current evidence suggests that enzalutamide is not significantly different from abiraterone with regard to improving the OS of mCRPC patients, but it has a greater effect on TTPP and rPFS. The evidence from this study can be used when selecting a treatment option for mCRPC in clinical practice. Due to the lack of a direct comparison, the conclusions drawn from the results of the indirect comparison performed in this analysis need to be verified in high-quality prospective studies.

## Data Availability Statement

The original contributions presented in the study are included in the article/supplementary material. Further inquiries can be directed to the corresponding author.

## Author Contributions

HG and ZW conceived this study, conducted the searching, and drafted the manuscript. CC and BL participated in article screening and performed the statistical analysis. CC, BL, and YL checked the data. HG and ZW contributed to the design of this study and provided proposals for the manuscript. All authors contributed to the article and approved the submitted version.

## Funding

This study was funded by Inner Mongolia Medical University and did not receive external funding.

## Conflict of Interest

The authors declare that the research was conducted in the absence of any commercial or financial relationships that could be construed as a potential conflict of interest.

## Publisher’s Note

All claims expressed in this article are solely those of the authors and do not necessarily represent those of their affiliated organizations, or those of the publisher, the editors and the reviewers. Any product that may be evaluated in this article, or claim that may be made by its manufacturer, is not guaranteed or endorsed by the publisher.

## Abbreviations

mCRPC, metastatic castration-resistant prostate cancer; TTPP, time to prostate-specific antigen progression; OS, overall survival; rPFS, radiographic progression-free survival; HR, hazard ratio; CI, confidence interval.
